# Commonly prescribed β-lactam antibiotics induce *C. trachomatis* persistence/stress in culture at physiologically relevant concentrations

**DOI:** 10.3389/fcimb.2014.00044

**Published:** 2014-04-11

**Authors:** Jennifer Kintner, Dawn Lajoie, Jennifer Hall, Judy Whittimore, Robert V. Schoborg

**Affiliations:** ^1^Department of Biomedical Sciences, Quillen College of Medicine, East Tennessee State UniversityJohnson City, TN, USA; ^2^Department of Pathology, Quillen College of Medicine, East Tennessee State UniversityJohnson City, TN, USA

**Keywords:** *Chlamydia trachomatis*, β-lactam, penicillin, antibiotic susceptibility, chlamydial persistence, chlamydial stress response, stressed chlamydiae

## Abstract

*Chlamydia trachomatis*, the most common bacterial sexually transmitted disease agent worldwide, enters a viable, non-dividing and non-infectious state (historically termed persistence and more recently referred to as the chlamydial stress response) when exposed to penicillin G in culture. Notably, penicillin G-exposed chlamydiae can reenter the normal developmental cycle upon drug removal and are resistant to azithromycin-mediated killing. Because penicillin G is less frequently prescribed than other β-lactams, the clinical relevance of penicillin G-induced chlamydial persistence/stress has been questioned. The goal of this study was to determine whether more commonly used penicillins also induce *C. trachomatis* serovar E persistence/stress. All penicillins tested, as well as clavulanic acid, induced formation of aberrant, enlarged reticulate bodies (RB) (called aberrant bodies or AB) characteristic of persistent/stressed chlamydiae. Exposure to the penicillins and clavulanic acid also reduced chlamydial infectivity by >95%. None of the drugs tested significantly reduced chlamydial unprocessed 16S rRNA or genomic DNA accumulation, indicating that the organisms were viable, though non-infectious. Finally, recovery assays demonstrated that chlamydiae rendered essentially non-infectious by exposure to ampicillin, amoxicillin, carbenicillin, piperacillin, penicillin V, and clavulanic acid recovered infectivity after antibiotic removal. These data definitively demonstrate that several commonly used penicillins induce *C. trachomatis* persistence/stress at clinically relevant concentrations.

## Introduction

The most common bacterial sexually transmitted disease (STD) agent in humans is *Chlamydia trachomatis* (serovars D-K), with 1,412,791 reported cases in the US in 2011 (Centers for Disease Control and Prevention, 2011). *C. trachomatis* genital infection is often chronic in women, with manifestations ranging from mild infection to infertility and ectopic pregnancy (Schachter, 1999). Most women with chlamydia-induced cervicitis are asymptomatic (Schachter et al., 1983) and at higher risk for serious complications. Genital co-infections with *Neisseria gonorrhoeae* and *C. trachomatis* infections are also frequent; in fact, *C. trachomatis* is historically the most common cause of post-gonococcal urethritis and cervicitis (PGU/PGC). Azithromycin and tetracycline/doxycycline are currently the treatments of choice for *C. trachomatis* infections in adults, though amoxicillin remains a recommended treatment for infected, pregnant women (Centers for Disease Control and Prevention, 2010).

*C. trachomatis* is a Gram-negative, obligate intracellular bacterium with a biphasic developmental cycle. The infectious, extracellular form (the elementary body or EB) enters host genital epithelial cells within an endosome. Following fusion of EB-containing endosomes, EB (0.2 μm diameter) develop into larger (0.8 μm), replicative, non-infectious reticulate bodies (RB). RB use ATP and metabolites from the host cell to grow and subsequently divide within a cytoplasmic inclusion. After 30–70 h, the RB mature into infectious EB and are released from the infected host cell (Wyrick, 2000).

It has become clear that the “biphasic” view of chlamydia development is incomplete. Exposure to certain adverse conditions can divert the developmental cycle into a state referred to as persistence or, more recently, as the chlamydial stress response. In persistence/stress, the chlamydiae are viable, but non-infectious (reviewed in Hogan et al., 2004; Wyrick, 2010; Schoborg, 2011). Penicillin G-exposure is one stressor that induces *C. trachomatis* to enter this state in culture. Penicillin G-exposed chlamydiae can remain persistent/stressed for up to 9 months and, upon antibiotic removal, can re-enter the normal developmental cycle (Galasso and Manire, 1961; Matsumoto and Manire, 1970). Persistent/stressed chlamydial inclusions contain enlarged RBs (abberent bodies or AB), and few infectious EBs (Matsumoto and Manire, 1970). Persistent/stressed chlamydiae are also viable, as indicated by: (i) continued synthesis of genomic DNA and unprocessed 16S rRNA; and (ii) their ability to re-enter the developmental cycle. However, they do not divide, are non-infectious, and less metabolically active (reviewed in Hogan et al., 2004; Wyrick, 2010; Schoborg, 2011). Published data also indicate that chlamydiae also enter this altered developmental state *in vivo*. Continued chlamydial infections and repeat infections with the same serovar are common, despite appropriate antibiotic therapy (Patton et al., 1994; Fortenberry et al., 1999; Dean et al., 2000). Chlamydial AB have been observed in samples from patients and infected animals (Nanagara et al., 1995; Skowasch et al., 2003; Pospischil et al., 2009; Rank et al., 2011). Finally, viable but non-infectious organisms are observed in the genital tracts of amoxicillin-treated, *C. muridarum*-infected mice (Phillips-Campbell et al., 2012).

Although the mechanism by which penicillins exert their anti-chlamydial effects has been controversial, *C. trachomatis* does express 3 penicillin-binding proteins (PBPs) (Barbour et al., 1982; Storey and Chopra, 2001). More recent data suggest penicillin G, mecillinam and piperacillin may inhibit chlamydial cell division by binding Pbp2 and Pbp3/FtsI (Ouellette et al., 2012). However, penicillin G and amoxicillin are the only β-lactams demonstrated to render chlamydiae persistent/stressed using all generally accepted critera (Galasso and Manire, 1961; Matsumoto and Manire, 1970; Phillips-Campbell et al., 2012). Ampicillin exposure reduces chlamydial infectivity and alters inclusion morphology (Johnson and Hobson, 1977; Beale et al., 1991; Wolf et al., 2000; Storey and Chopra, 2001), while mecillinam and piperacillin induce AB formation (Storey and Chopra, 2001; Ouellette et al., 2012), suggesting induction of chlamydial persistence/stress. However, the first indication that β-lactams might not be lethal for developing chlamydiae came from clinical observations in the 1960s (Holmes et al., 1967; Richmond et al., 1972).These authors reported frequent cases of *C. trachomatis*-mediated PGU/PGC in patients several months following successful eradication of *N. gonorrhoeae* with penicillin therapy. As a result, in most cases, penicillins are not considered front-line anti-chlamydial drugs.

## Materials and methods

### Chlamydia and host cells

A human urogenital isolate of *C. trachomatis* E/UW-5/CX was obtained from S. P. Wang and C. C. Kuo (University of Washington, Seattle, WA). This strain was propagated in McCoy cells and used for all experiments. HeLa cells, a human cervical adenocarcinoma epithelial cell line (ATCC No. CCL2), were cultivated in growth medium (Minimal Essential Medium (MEM) with Earle's salts containing L-glutamine, 10% fetal calf serum (Atlanta Biologicals) and 1 μg/mL gentamicin) at 37°C and 5% CO_2_ on standard tissue culture plates or glass coverslips.

### Chlamydial infection and antibiotic exposure

Host cell monolayers were either mock-infected (exposed to 2SPG: 0.2 M sucrose, 6 mM NaH_2_PO_4_, 15 mM Na_2_HPO_4_, 5 mM L-glutamine, pH 7.2) or *C. trachomatis*-infected at a multiplicity of infection (MOI) of 1, using crude EB stock diluted in 2SPG for 1 h at 35°C. Monolayers used for minimal inhibitory concentration (MIC)/immunofluorescent (IFA) or minimal bactericidal concentration (MBC) analyses were then immediately refed with growth medium plus diluent (ddH_2_O) or the antibiotic of interest and incubated for 54 h at 35°C. Monolayers used in all other antibiotic-exposure experiments were first refed with medium and incubated at 35°C for 24 h, after which the supernatant was replaced with medium + diluent or the antibiotic of interest. Cultures were then harvested for analysis at 30 h post-antibiotic addition, a total of 54 h post-infection (hpi). In recovery experiments, replicate antibiotic-exposed cultures were washed at 54 hpi, refed with either antibiotic-replete (non-recovered group) or antibiotic-deficient (recovered group) medium, and incubated at 35°C for 3 additional days (a total of 126 hpi).

### Fluorescent and transmission electron microscopy

IFA and high-contrast transmission electron microscopy (TEM) analyses were performed as described (Deka et al., 2006). Fluorescent photomicrographs were taken using a Zeiss Axiovert S100 microscope/Axiocam camera, and converted to grayscale using Adobe Photoshop V5.0.

### Chlamydial infectivity assay

Production of infectious *C. trachomatis* EB was assayed using a subpassage titer assay as described (Deka et al., 2006), except that phosphonoformate was omitted from the medium. The number of inclusion forming units (IFU)/mL in the undiluted inoculum was calculated from triplicate determinations.

### RNA and DNA isolation

Total RNA and DNA were isolated simultaneously from experimental samples using the RNeasy Mini (Qiagen) and QIAmp DNA Blood Mini (Qiagen) kits as described (Deka et al., 2006). Total RNA and DNA preparations were quantified using optical density (OD) at 260 and 280 nm; all samples had OD260/280 ratios >1.9. RNA sample quality was assessed by using a 2100 Bioanalyzer (Agilent) and the RNA 6000 Nano LabChip kit. All samples had RNA Integrity Numbers (RINs) >9.0.

### Reverse transcription, PCR, and RT-PCR

Reverse transcription of total RNA, PCR, and RT-PCR were performed as described (Deka et al., 2006). PCR and RT-PCR was performed using purified total cellular DNA or cDNA as templates, respectively. Control and experimental templates were diluted from 1/10 to 1/1000 in ddH_2_O to insure that each reaction was quantified in the linear amplification range. Primer sets used to amplify the human glyceraldehyde-3-phosphate dehydrogenase (GAPDH) and chlamydial 16S rRNA genes, as well as for *C. trachomatis* unprocessed 16S rRNA transcript have been published (Deka et al., 2006). After PCR, all reaction products were electrophoresed, quantified and normalized as described (Deka et al., 2006).

### Statistical analyses

Statistics were performed using Microsoft Excel. Means were compared by 2-sample t-test for independent samples. *P* = 0.05 were considered significant. All plotted values are averages of three biological replicates ± standard error of the mean (s.e.m.) and each experiment was performed three times independently.

## Results

### Rationale for antibiotic-exposure conditions

Though penicillin G is a characterized chlamydial persistence inducer, it is less widely used than other β-lactams. Also, as penicillins are no longer recommended for anti-chlamydial therapy in adults, one might question the relevance of penicillin-induced chlamydial persistence *in vivo*. It is important to recognize that penicillins are commonly utilized to treat other bacterial infections—with the result that developing chlamydiae within asymptomatically-infected individuals are exposed to β-lactams during therapy for other concurrent infections. Thus, the penicillins evaluated in this study were chosen primarily according to prescription frequency: amoxicillin (AMX), clavulanic acid (CLA), ampicillin (AMP), and penicillin V (PEN V) were all among the top 200 most prescribed drugs in the US in 2011 (Bartholow, 2011). Carbenicillin (CAR) and piperacillin (PIP) were chosen because of their high efficacy for Gram-negative bacteria. Aztreonam (ATM), a monobactam, was chosen because of its structural dissimilarity to penicillin. The cephalosporin ceftriaxone (CRO) was chosen because it is a recommended treatment for gonococcal infections (Centers for Disease Control and Prevention, 2010), which often occur simultaneously with *C. trachomatis*. Finally, cefotaxime (CTX) was chosen to confirm any effect of CRO on chlamydial development. The standard concentration of each drug used (denoted as 1X in Table 1) was equivalent to the serum concentration obtained after administration of a standard dose in clinical trials (McEvoy, 2004), increasing the *in vivo* relevance of any observed effects.

**Table 1:** **The 1X concentrations, MIC and MBC of all drugs used in this study**.

**Drug**	**Class**	Serum (1X)^*^ concentration	MIC^**^	MBC^***^
Amoxicillin (AMX)	Penicillin	11 μ g/mL	>1100 μg/mL	0.011 μ g/mL
Ampicillin (AMP)	Penicillin	3.7 μ g/mL	>370 μ g/mL	0.037 μ g/mL
Aztreonam (ATM)	Monobactam	1.8 μ g/mL	>180 μ g/mL	>180 μ g/mL
Carbenicillin (CAR)	Penicillin	1.9 μ g/mL	>190 μ g/mL	0.019 μ g/mL
Cefotaxime (CTX)	Cephalosporin	2.53 μ g/mL	>253 μ g/mL	>253 μ g/mL
Ceftriaxone (CRO)	Cephalosporin	0.93 μ g/mL	>93 μ g/mL	>93 μ g/mL
Clavulanic acid (CLA)	β-lactamase inhibitor	3.5 μ g/mL	>350 μ g/mL	0.35 μ g/mL
Penicillin G (PEN G)	Penicillin	20 U/mL	>2000 U/mL	0.02 U/mL
Penicillin V (PEN V)	Penicillin	0.2 μ g/mL	>20 μ g/mL	0.002 μ g/mL
Piperacillin (PIP)	Penicillin	39 μ g/mL	>3900 μ g/mL	0.39 μ g/mL
Tetracycline (TET)	Tetracycline	4.3 μ g/mL	0.043 μ g/mL	ND

* *The 1X concentration was defined as the peak serum concentration of each drug obtained in clinical trials after administration of a standard dose (McEvoy, 2004)*.

** *The MIC was defined as the minimal concentration of each drug required to prevent formation of chlamydial inclusions. In most cases, a precise MIC could not be determined because the highest tested drug concentration (100X) did not prevent inclusion formation*.

*** *The MBC was defined as the minimal concentration of each drug required to reduce infectious EB production in sub-titer assays by >99%. In the case of ATM, CTX, and CRO, MBC could not be determined because the highest tested drug concentration (100X) did not reduce infectious titer by >99%*.

### No penicillin of interest inhibits inclusion formation

**Figure 1:**
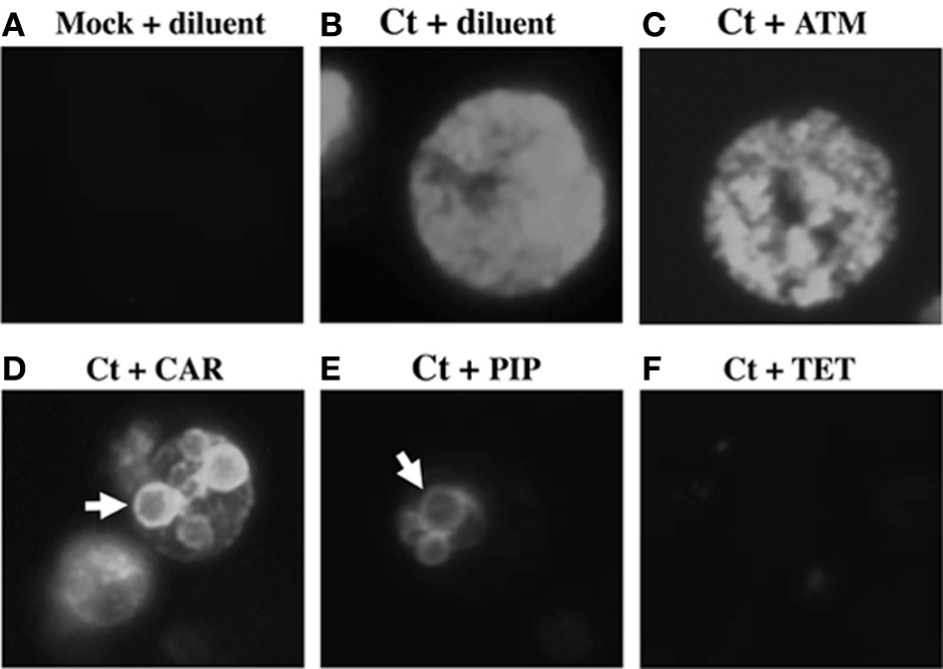
**Penicillin-exposure alters chlamydial morphology**. HeLa cells were plated on glass coverslips, infected with *C. trachomatis* and refed immediately post-infection with growth medium + various dilutions of each antibiotic of interest. At 54 hpi, infected cells were fixed and stained with FITC-conjugated anti-*C. trachomatis* MOMP as described. **(A)** Mock-infected cells + ddH_2_O (diluent). **(B)** *C. trachomatis*-infected (Ct) cells + diluent. **(C)** *C. trachomatis* + ATM. **(D)** *C. trachomatis* cells + CAR. **(E)** *C. trachomatis* + PIP. **(F)** *C. trachomatis* + TET. Panels **(B–E)** are representative inclusions photographed at 1000X magnification. Inclusions in panels **(C–F)** were drug-exposed at the 1X concentration (Table 1).

To determine whether the antibiotics of interest blocked inclusion formation or EB production, chlamydia-infected cells were exposed to 10-fold antibiotic dilutions from 1 to 54 hpi. MIC for the chlamydiae has been variously defined by either inhibition of inclusion formation (Welsh et al., 1992) or by abnormal inclusion morphology (Storey and Chopra, 2001). Because identification of “abnormal inclusions” seemed subjective, we defined the MIC as the minimal drug concentration that prevented inclusion development (Welsh et al., 1992). MBC was defined as the drug concentration required to reduce infectious EB production by >99% (Welsh et al., 1992). As no penicillin tested inhibited inclusion formation, even at 100 times the serum concentration, MICs could not be calculated (Table 1). In contrast, the MIC for tetracycline (TET) was 0.043 μ g/mL (Table 1), similar to published values (Welsh et al., 1992). Exposure to all 6 penicillins tested, as well as to CLA, produced small inclusions containing large, spherical structures, similar to AB formed during persistence/stress (Figures 1D,E, white arrows). In contrast, diluent- (ddH_2_O), ATM- and cephalosporin-exposed inclusions were indistinguishable from each other (Figures 1B,C and data not shown). Finally, mock-infected controls contained no inclusions (Figure 1A) and TET-exposure prevented inclusion formation (Figure 1F), as expected. Thus, all penicillins tested altered inclusion morphology, but did not prevent inclusion development.DNA and unprocessed

### Penicillin-exposure induces RB morphologic alterations characteristic of persistence/stress

**Figure 2:**
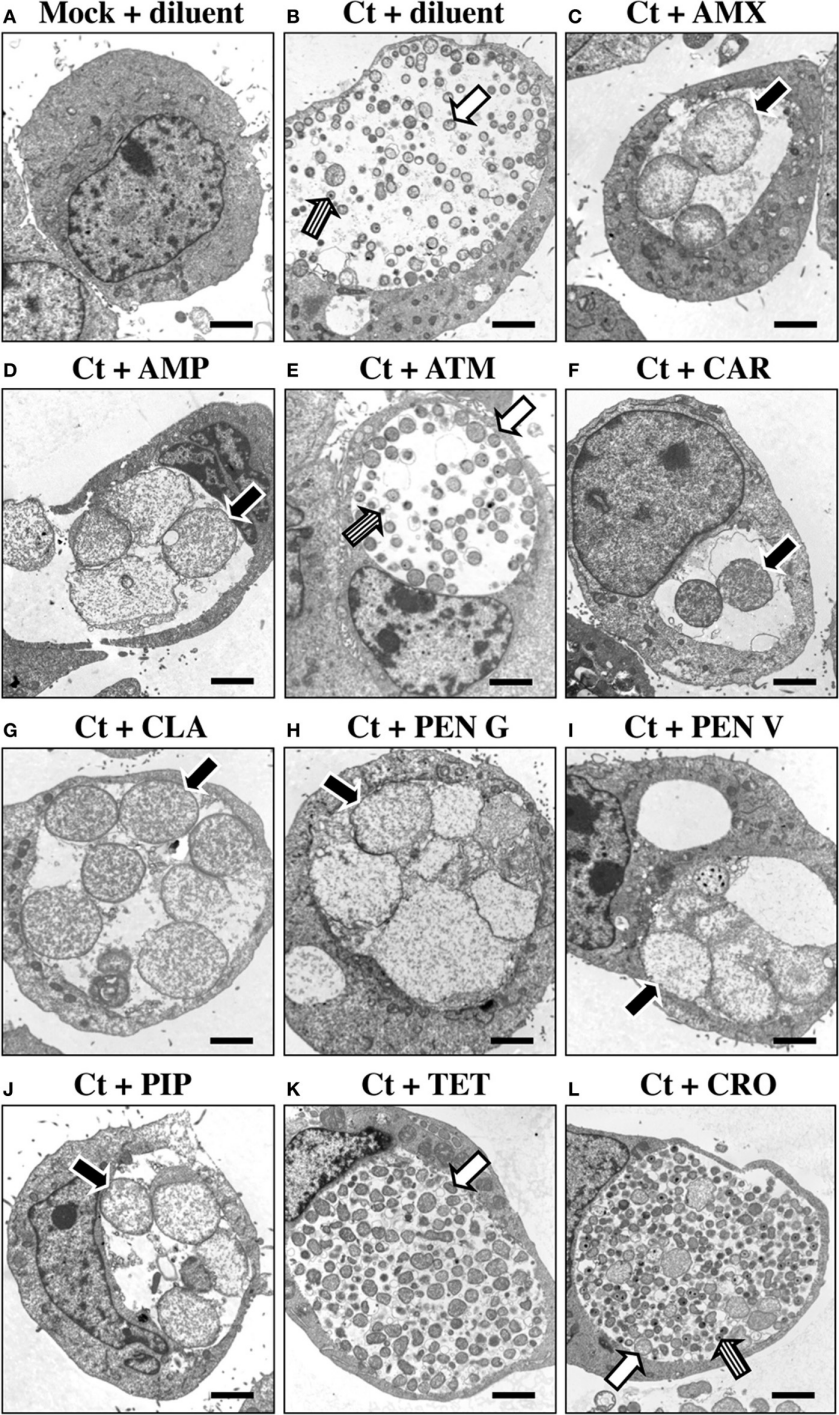
**Penicillin-exposure induces chlamydial AB formation**. HeLa cells were *C. trachomatis*-infected and incubated in the absence of antibiotic for 24 h. Infected and uninfected cultures were then refed with medium containing each antibiotic of interest at the 1X concentrations (Table 1). Cells were incubated for an additional 30 h (a total of 54 hpi), fixed and subjected to TEM. **(A)** Mock-infected cells + ddH_2_O (diluent). **(B)** *C. trachomatis*-infected (Ct) cells + diluent. **(C)** *C. trachomatis* + AMX. **(D)** *C. trachomatis* + AMP. **(E)** *C. trachomatis* + ATM. **(F)** *C. trachomatis* + CAR. **(G)** *C. trachomatis* + CLA. **(H)** *C. trachomatis* + PEN G. **(I)** *C. trachomatis* + PEN V. **(J)** *C. trachomatis* + PIP. **(K)** *C. trachomatis* + TET. **(L)** *C. trachomatis* + CRO. Morphologically normal RB and EB are indicated by white and striped arrows respectively. Abberent bodies (AB) are labeled with black arrows. Each photomicrograph is at 7500X magnification; the black bar at the lower right of each panel represent 2 μm.

PEN G-exposed, persistent/stressed chlamydial RB (termed AB) have a distinctive morphology (Matsumoto and Manire, 1970). To determine whether exposure to other β-lactams induces similar structural alterations, *C. trachomatis*-infected HeLa cells were antibiotic-exposed from 24 to 54 hpi and subjected to TEM. This exposure time was chosen to mimic an *in vivo* situation in which chlamydiae are penicillin-exposed after they have initiated development. Importantly, *C. trachomatis* serovar E under identical culture conditions is primarily in the RB stage, and still susceptible to the effects of penicillin G, up to 44 hpi (Deka et al., 2006, 2007). Inclusions in *C. trachomatis*-infected, diluent-exposed cells contained normal RB (white arrow) as well as intermediate bodies (IB) and EB (striped arrow; Figure 2B). ATM-exposed chlamydiae (Figure 2E) were indistinguishable from diluent-exposed controls. Though occasional AB were observed in CRO- and CTX-exposed cultures, most RB were normal in appearance and abundant EB were observed (Figure 2L and data not shown, white and striped arrows respectively). In contrast, AMX- (Figure 2C), AMP- (Figure 2D), CAR- (Figure 2F), PEN V- (Figure 2I), PIP- (Figure 2J), and CLA- (Figure 2G) exposed chlamydial inclusions contained swollen, irregular AB (black arrows), and few IBs or EBs were observed. PEN G-exposed chlamydiae were similar morphologically to those exposed to the other penicillins (Figure 2H), and, as expected, were characteristic of persistent chlamydiae (Matsumoto and Manire, 1970). Finally, though few IB or EB were observed in TET-exposed cultures, AB were not observed (Figure 2K, white arrow). These observations indicate that exposure to commonly prescribed β-lactam antibiotics, as well to CLA, induce formation of chlamydial AB.

### Penicillins reduce production of infectious EBs at physiologically-relevant concentrations

**Figure 3:**
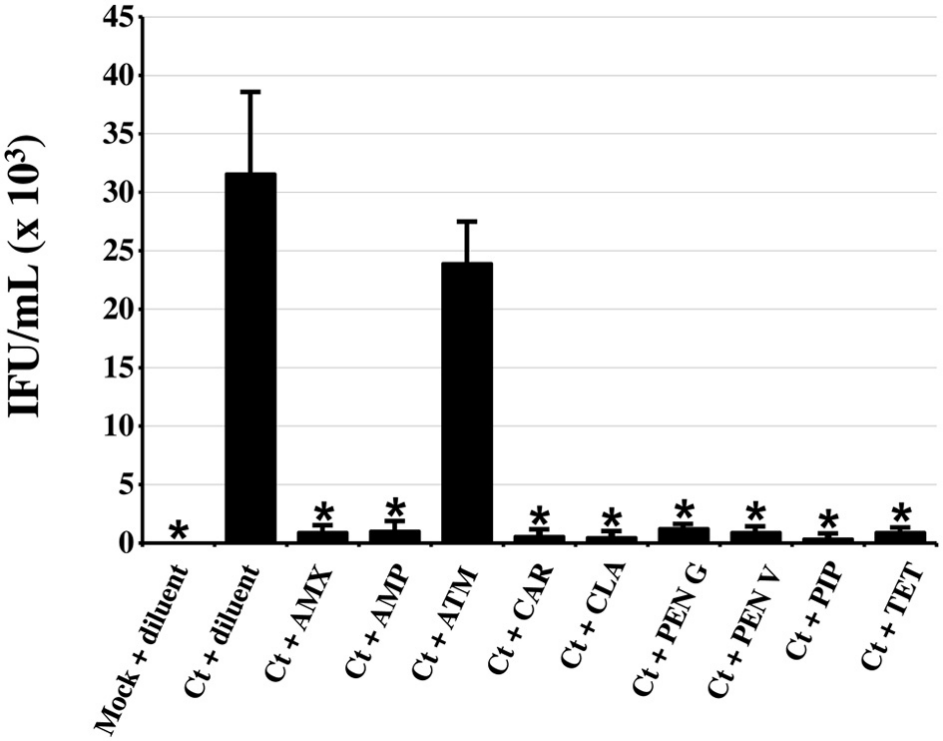
**Penicillin-exposure significantly reduces chlamydial infectivity**. HeLa cells were *C. trachomatis*-infected and incubated in the absence of antibiotic for 24 h. Infected and uninfected cultures were then refed with medium containing each antibiotic of interest at the 1X concentrations (Table 1). Thirty hours post-antibiotic addition (54 hpi total), infected cell lysates were collected and used for infectious titer analyses. Inclusion counts were averaged and used to calculate inclusion forming units (IFU)/mL. The average of three biologic replicates from one of three independent experiments ± s.e.m. is shown. Groups significantly different from the diluent-exposed, Ct-infected control are indicated by asterisks (^*^), *P* ≤ 0.05 was considered significant.

Persistent/stressed chlamydiae, while viable, are non-infectious (reviewed in Hogan et al., 2004; Wyrick, 2010; Schoborg, 2011). TEM studies indicated that penicillin-exposed, *C. trachomatis*-infected cells contained few EB (Figure 2), suggesting that production of infectious organisms was inhibited by all the penicillins of interest. To confirm these observations, chlamydiae-infected HeLa monolayers were exposed to each antibiotic from 24 to 54 hpi and subjected to sub-passage titration analyses (Figure 3). As expected, PEN G-exposure significantly reduced chlamydial infectivity. Likewise, exposure to AMX, AMP, CAR, PEN V, PIP, and CLA at the 1X serum concentration reduced production of infectious *C. trachomatis* EB by >95% compared to the diluent-exposed, chlamydiae-infected controls. In contrast, neither ATM- (Figure 3) nor cephalosporin-exposure (data not shown) significantly reduced chlamydial infectivity. As expected, production of infectious EB was essentially abolished by TET-exposure. These data demonstrate that exposure to penicillins or CLA significantly reduces production of infectious EB from infected cells.

### Penicillin-exposure does not reduce accumulation of either *C. trachomatis* genomic DNA or unprocessed 16s rRNA

**Figure 4:**
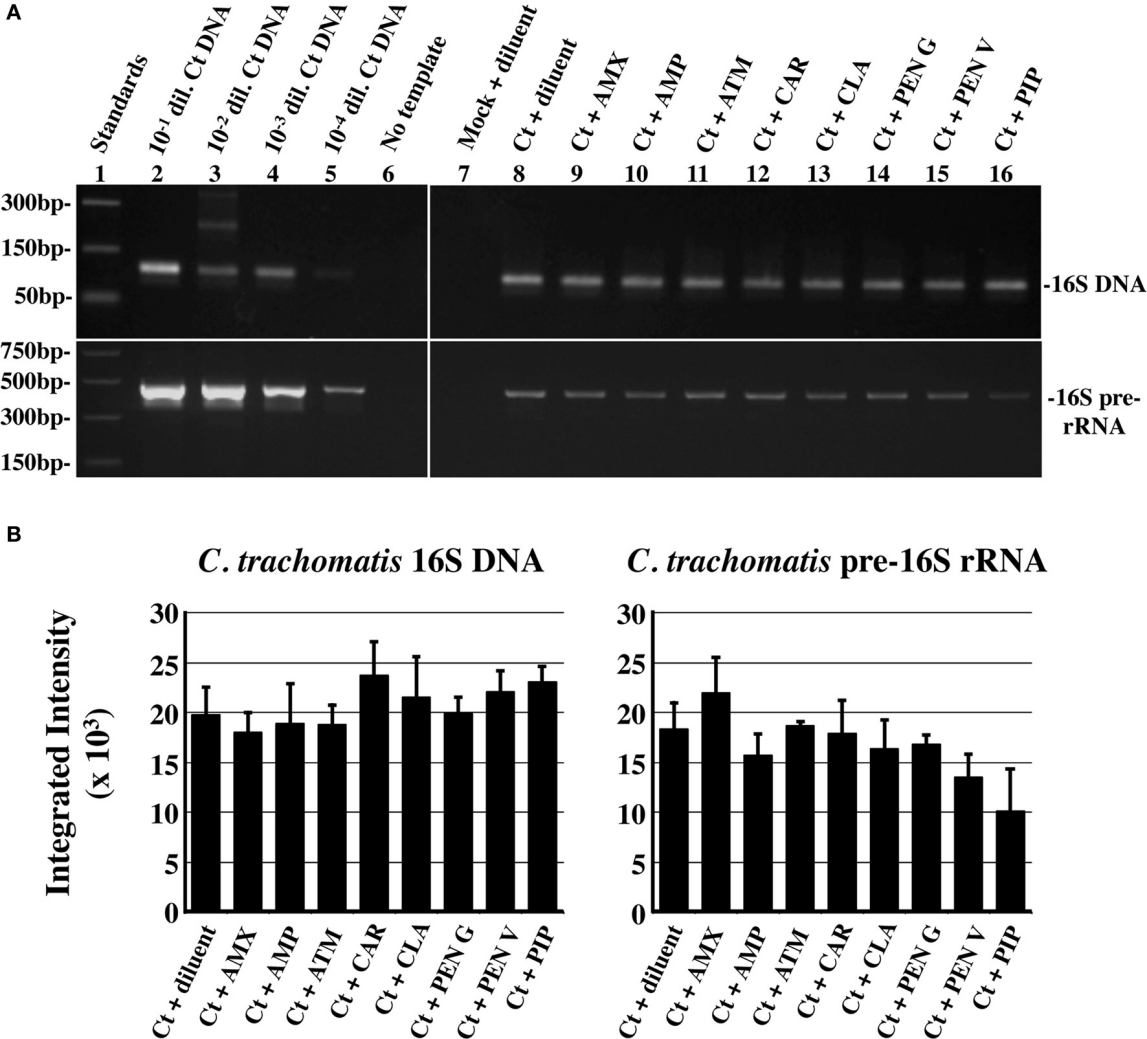
**Penicillin-exposure does not reduce genomic DNA or pre-16S rRNA accumulation**. Total DNA and RNA from 1X antibiotic-exposed, infected cells was subjected to semi-quantitative PCR (or RT-PCR) using primers specific for human GAPDH (DNA), chlamydial 16S rRNA (DNA), and chlamydial unprocessed 16S rRNA transcripts (RNA). **(A)** Representative PCR gel images. Amplification of + control DNA dilutions are shown to the left. **(B)** Plots of chlamydial genomic DNA amplimer quantity normalized to host genomes (left) and pre-ribosomal RNA-specific amplimer quantity normalized to chlamydial genomes (right). The average of three independent biologic replicates from one of three independent experiments ± s.e.m. is shown. None of the experimental groups were significantly different from the diluent-exposed, infected control at *P* ≤ 0.05.

Though non-infectious, persistent/stressed chlamydiae remain viable and continue to synthesize (and accumulate) both genomic DNA and unprocessed 16s rRNA (Gerard et al., 1998, 2001). To assess chlamydial viability, chlamydiae-infected, HeLa monolayers were exposed to each antibiotic under the conditions described above. Host GAPDH DNA, chlamydial 16S DNA and chlamydial unprocessed 16S rRNA were quantified as described (Deka et al., 2006) and representative gels used for quantification of relative *C. trachomatis* 16S DNA and unprocessed 16S rRNA accumulation are shown in Figure 4A. Statistical analysis (Figure 4B) indicated that there was no significant reduction in either chlamydial genome accumulation or unprocessed 16S rRNA accumulation in any of the antibiotic-exposed, infected cultures compared to that in diluent-exposed, infected cells. These data are consistent with previous observations that *C. trachomatis* L2 genomic DNA accumulation is unaffected by PEN G-exposure (Lambden et al., 2006). They also suggest that penicillin-exposed chlamydiae continue chromosomal and unprocessed 16S rRNA accumulation and are, therefore, viable.

### Penicillin-exposed chlamydiae resume production of infectious EB after drug removal

**Figure 5:**
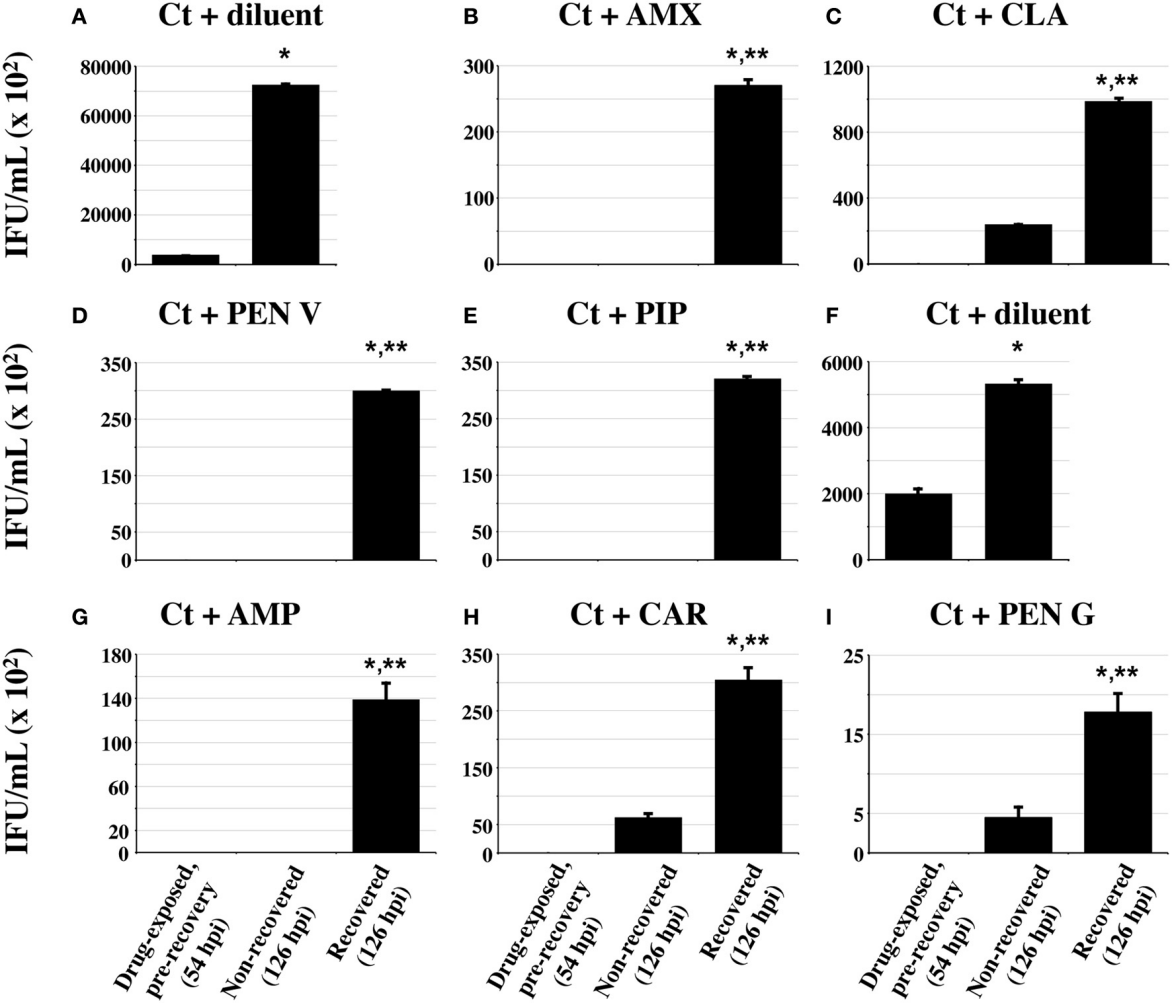
**Penicillin-induced stress/persistence is reversible**. Replicate cultures of HeLa cells were infected and antibiotic-exposed at concentrations 10-fold higher than the MBC for each drug (Table 1) using the timing scheme described in Figure 3. At 30 h post-antibiotic addition (54 hpi total), one set of cultures was harvested for “pre-recovery” EB titration as in Figure 3. Duplicate antibiotic-exposed and control cultures were washed, refed with either antibiotic-replete (non-recovered samples) or antibiotic-deficient (recovered samples) growth medium and allowed to recover for 3 additional days (a total of 126 hpi). These cultures were then processed for EB titration. Note that each drug-exposure experiment was divided into two separate sets. The diluent-exposed control for panels **(B–E)** is shown in panel **(A)** and the diluent-exposed control for panels **(G–I)** is shown in panel **(F)**. The average of three biologic replicates ± s.e.m. is shown and *p* ≤ 0.05 was considered significant. Single asterisks (^*^) indicate titers that are significantly higher than those observed in the pre-recovery cultures within each drug-exposure group. Double asterisks (^**^) denote titers that are significantly increased compared to the non-recovered cultures within each group. The experiment shown is one of three independent experiments.

One important characteristic of persistent/stressed chlamydiae is that they can re-enter the normal developmental cycle and produce infectious EB when the stressor is removed (reviewed in Hogan et al., 2004; Wyrick, 2010; Schoborg, 2011). Therefore, antibiotic recovery experiments were carried out as described in the methods. As expected, the yield of infectious chlamydiae immediately after the initial 30 h antibiotic-exposure (Figures 5B–E,G–I) was much lower than that from diluent-exposed, infected controls (Figures 5A,F). Chlamydial titers obtained after 3 days of additional antibiotic-exposure (“non-recovered” cultures) remained low in all cases. In contrast, significantly increased chlamydial titers were observed in AMX-, AMP-, CAR-, PEN V-, PIP-, and CLA-exposed cultures after the 3 day recovery period (recovered samples), compared to those obtained either immediately after initial drug-exposure or from parallel “non-recovered” cultures (Figures 5B–E,G,H). As expected, PEN G-exposed chlamydiae recovered infectivity after drug removal as well (Figure 5I). These data indicate that chlamydiae exposed to commonly prescribed penicillins can recover infectivity when the drugs are removed. Notably, the total amount of infectivity recovered after antibiotic removal was different for each drug tested.

## Discussion

AMX-, AMP-, CAR-, PEN V-, PIP-, and CLA-exposed chlamydiae exhibit defining characteristics of persistent/stressed organisms (reviewed in Hogan et al., 2004; Wyrick, 2010; Schoborg, 2011). They are viable (as evidenced by continued genome and pre-16S rRNA accumulation and the ability to recover infectivity after drug removal) but non-infectious (as shown by reduced chlamydial titer). These drugs also induce AB formation, which is consistent persistence/stress induction (Matsumoto and Manire, 1970). Thus, these commonly prescribed β-lactams induce chlamydial persistence/stress in culture at physiologically-relevant concentrations. The CDC currently recommends either azithromycin or AMX for treatment of chlamydia-infected, pregnant women (Centers for Disease Control and Prevention, 2010). However, with recent reports linking azithromycin to adverse cardiac outcomes (Ray et al., 2012), more physicians may elect to use AMX in this situation. Whether or not AMX-treated, *C. trachomatis*-infected women have a higher risk of subsequent tubal factor infertility is currently unknown. However, because: (i) chlamydiae can recover from AMX-induced persistence/stress in culture; and (ii) resumption of shedding is observed after AMX-treatment cessation in chlamydia-infected mice (Phillips-Campbell et al., 2012), it seems reasonable to conclude that AMX-treated, infected women may remain at risk for chronic infection and reproductive complications. Thus, if AMX is used, it is important that chlamydial eradication be confirmed post-therapy, as per CDC recommendations (Centers for Disease Control and Prevention, 2010).

CLA, a β-lactam originally isolated from *Streptomyces clavuligerus*, is a strong β-lactamase inhibitor and is used clinically to increase β-lactam activity against penicillinase-producing bacterial strains (Reading and Cole, 1977). CLA also has direct antimicrobial activity, albeit weaker than that of other β-lactams. Interestingly, the reported CLA MBC for *E. coli* is 25 μ g/mL (Neu and Fu, 1978), more than 70 times higher than that for *C. trachomatis* (Table 1). Because CLA binds *E. coli* PBP2 (Spratt et al., 1977) and *C. trachomatis* expresses a PBP2 homolog that may function in RB division (Ouellette et al., 2012), it seems likely that CLA induces chlamydial persistence/stress by inhibiting PBP2 function. This would be consistent with published observations that the PBP2-specific drug, mecillinam, also induces AB formation and reduces EB production (Storey and Chopra, 2001; Ouellette et al., 2012). Our data (Table 1) also indicate that *C. trachomatis* serovar E is resistant to ATM, CTX and CRO, at least at physiologically achievable concentrations.

Approximately 50% of total antibiotics used in human medicine are β-lactams, most of which are aminopenicillins - like AMX and AMP (Kümmerer and Henninger, 2003; Goossens et al., 2005). Since there are about 1.5 million reported new chlamydial infections per year in the US (Centers for Disease Control and Prevention, 2011), up to 75% of which are asymptomatic (Detels et al., 2011), brief episodes of β-lactam-induced persistence/stress may occur in many patients during treatment for other bacterial infections. There are also other routes by which chlamydia-infected hosts could be chronically exposed to low levels of β-lactam and other antibiotics. For example, there is widespread, low-level β-lactam contamination of milk, meat and other agricultural products. The risks posed by such contamination, most notably penicillin allergy and promotion of antibiotic resistance, have been recognized for decades (Welch, 1956). Antibiotics and their degradation products (DPs) are also found in ground water, the most commonly identified of which are macrolides, sulfonamides, fluoroquinalones and tetracyclines (Heberer, 2002; Kolpin et al., 2002). Notably, exposure to low concentrations of erythromycin (a macrolide), ciprofloxacin and ofloxacin (both fluoroquinalones) also induces persistence/stress in both *C. pneumoniae* and *C. trachomatis* (Dreses-Werringloer et al., 2000, 2001; Gieffers et al., 2004). Though most β-lactams are rapidly converted to DPs by UV light and chlorination, some AMX-DPs, like AMX S-oxide, are environmentally stable and retain an intact β-lactam ring (Gozlan et al., 2010). Therefore, DPs of β-lactams or other antibiotics consumed in treated water could induce chlamydial persistence/stress *in vivo*. However, the effect of antibiotic DPs on chlamydial development, if any, is currently unknown.

Treatment failure is a significant problem in human chlamydial infections (Horner, 2006; Pitt et al., 2013). For example, post-treatment *C. trachomatis* positivity is reported in 10-15% of women on recommended treatment regimens (Wang et al., 2005). Significantly, penicillin G-exposed, persistent/stressed *C. trachomatis* serovar E is resistant to azithromycin killing in culture (Wyrick and Knight, 2004). Likewise, IFN-exposed, persistent/stressed *C. trachomatis* is more resistant to doxycycline (Reveneau et al., 2005). Azithromycin and ofloxacin also do not eradicate persistent/stressed *C. pneumoniae* in culture (Kutlin et al., 1999). Finally, induction of persistence/stress using AMX increases azithromycin treatment failure in *C. muridarum*-infected mice (Phillips-Campbell et al., 2014). These studies suggest that *in vivo* persistence/stress could be one mechanism by which chlamydiae resist antimicrobial therapy *in vivo*. Thus, given the widespread medical use of these antibiotics, as well as their presence in food and water, β-lactam-induced chlamydial persistence/stress may have significantly more *in vivo* relevance than previously assumed.

## Author contributions

Jennifer Kintner and Dawn Lajoie performed drug exposure, infectious titer, RT-PCR and recovery experiments; Jennifer Hall assisted with RT-PCR experiments, data analysis and manuscript preparation; Judy Whittimore performed all TEM experiments; and Robert V. Schoborg designed the study, assisted in titer and TEM experiments, analyzed data and prepared the manuscript. All authors have read and approved the manuscript.

### Conflict of interest statement

The authors declare that the research was conducted in the absence of any commercial or financial relationships that could be construed as a potential conflict of interest.
